# Supplementary material for: Citclops: A next-generation sensor system for the monitoring of natural waters and a citizens' observatory for the assessment of ecosystems’ status
Source: PLoS One. 2020 Mar 26;15(3):e0230084. doi: 10.1371/journal.pone.0230084 (PMC7098649; doi:10.1371/journal.pone.0230084)
Supplement: S1 Appendix — (DOCX) [file pone.0230084.s001.docx]

## ANNEX A – Required python libraries

| Library | Version |
| --- | --- |
| Flask | 0.10.1 |
| Jinja2 | 2.7.3 |
| MarkupSafe | 0.23 |
| Shapely | 1.5.5 |
| Werkzeug | 0.10.1 |
| argparse | 1.2.1 |
| distribute | 0.6.24 |
| h5py | 2.3.1 |
| itsdangerous | 0.24 |
| mglob | 0.4 |
| netcdf | 0.0.30 |
| numpy | 1.8.0 |
| psycopg2 | 2.6 |
| pyandoc | 0.0.1 |
| python-dateutil | 2.4.0 |
| requests | 2.5.3 |
| requests-cache | 0.4.9 |
| six | 1.9.0 |
| wsgiref | 0.1.2 |
| netCDF4 | 1.1.0 |
| gunicorn | 19.2.1 |
